# PROXIMITY, CARE VALUES, AND CAREGIVING STRAIN: EVIDENCE FROM THE 2020 NAC/AARP NATIONAL SURVEY

**DOI:** 10.1093/geroni/igae098.3884

**Published:** 2024-12-31

**Authors:** Tingan Jin

**Affiliations:** The London School of Economics and Political Science, London, England, United Kingdom

## Abstract

The concept of distant caregiving emerged as family caregivers can care for older adults in settings other than living with their parents. Previous studies have illustrated that differences in geographical distance could lead to different caregivers’ experiences in terms of physical, emotional, and financial strain. This study uses the most recent nationally representative data on caregivers in the US from the National Alliance for Caregiving and AARP to explore the characteristics and values of caregivers with varying distances to care recipients and test the effect of geographical distance on various aspects of caregiving strain. The preliminary analysis conducted in June 2024 suggests that caregivers providing care at a distance reported lower physical and financial strain levels regardless of proximity intervals. Long-distance carers, namely those who live more than one hour away from their parents, show more physical strain (b=-0.236, p< 0.05) and financial strain (b =-0.228, p< 0.05) than those who live with or close to their parents. Findings regarding emotional strain were against previous studies, and we found that while those who live close to their parents reported lower levels of emotional strain (b=-0.171, p< 0.05), the pattern does not apply to long-distance caregivers with a positive, statistically insignificant relationship. Adding components regarding caregivers’ willingness and perceptions towards care provision, it is found that whether the respondents feel they had a choice to take care of their parents is a statistically significant predictor of physical (b=0.508, p< 0.05), financial (b=0.446, p< 0.05), and emotional strain (b=0.810, p< 0.05).

